# Early Neonatal Mortality (< 24 h) in Ecuador: A Population-Based Study on the Impact of Apgar Score, Gestational Age, Birth Weight, Delivery Type, and Healthcare Level

**DOI:** 10.1155/ijpe/4225987

**Published:** 2025-05-19

**Authors:** Iván Dueñas-Espín, María Alejandra Montaluisa, Andrea Aguilar-Molina, Fernando Aguinaga, Luciana Armijos-Acurio, Ruth Jimbo-Sotomayor, Ángela León Cáceres, María F. Rivadeneira, Silvana Rivera-Guerra, Xavier Sánchez, Betzabé Tello

**Affiliations:** ^1^Instituto de Salud Pública, Facultad de Medicina, Pontificia Universidad Católica del Ecuador, Quito, Ecuador; ^2^School of Public Health, Imperial College London, London, UK; ^3^Postgrado de Pediatría, Facultad de Medicina, Pontificia Universidad Católica del Ecuador, Quito, Ecuador; ^4^Sociedad Ecuatoriana de Pediatría, Quito, Ecuador; ^5^Centro de Investigación Para la Salud en América Latina (CISeAL), Pontificia Universidad Católica del Ecuador, Quito, Ecuador; ^6^Heidelberg Institute of Global Health, Faculty of Medicine, University of Heidelberg, Heidelberg, Germany

**Keywords:** Apgar score, early neonatal mortality, health determinants, neonatal risk factors, premature infants

## Abstract

**Background:** We examined the relationship between the 5-min Apgar score and other individual and contextual neonatal characteristics with early neonatal mortality (before 24 h) in Ecuadorian neonates.

**Methods:** We conducted a retrospective case–control study using data from the Ecuadorian National Surveillance System for Neonatal Mortality, covering January 2014 to September 2017. We analyzed data for neonates who died within 28 days of birth, focusing on mortality before 24 h (early death). Multilevel multivariate logistic regression was used to calculate crude and adjusted odds ratios (aORs) for early death based on 5-min Apgar scores and other neonatal factors. The random effects variable in this model was altitude at which neonates were attended; this component of the model allows the intercept estimates to vary randomly among the groups of altitude, suggesting that there are differences between these groups that could influence the study results at which the neonates were attended.

**Results:** Of 2144 neonates analyzed, 53.2% were male, with an average gestational age of 30.8 weeks and a mean birth weight of 1525.2 g. More than half (56.0%) were delivered by caesarean section. Common comorbidities included prematurity (39.4%), asphyxia (31.2%), and infections (24.7%). Also, 17.4% had Apgar scores of ≤ 3. After adjustment, Apgar scores of ≤ 3 were linked to significantly increased odds of death before 24 h, with an aOR of 20.65 (95% CI: 5.99–71.28, *p* < 0.001).

**Conclusions:** This study demonstrated that the Apgar score, among other determinants, was significantly associated with early neonatal mortality (before the first 24 h). This association was independent of type of delivery, comorbidities, disorders related to asphyxia, prematurity, infections, and other medical disorders, as well as varying levels of care from primary to tertiary. These findings underscore the importance of Apgar evaluation in neonates and suggest a predictive value of the score for early neonatal mortality.

## 1. Introduction

In 1953, Dr. Virginia Apgar introduced the Apgar score as a straightforward, yet potent, instrument for assessing a neonate's adaptation and reaction to life outside of the womb [1]. The Apgar score evaluates five factors—muscle tone, heart rate, reflex response, skin color, and respiration—at 1 and 5 min postbirth, with further assessments if the 5-min score is below 7.

A 5-min Apgar score ranging from 7 to 10 is universally recognized as reassuring, a score of 4–6 indicates moderate anomaly, and a score between 0 and 3 is considered concerning for term and late-preterm infants [2, 3]. Unfortunately, several studies have cast doubt on the reliability of this categorization for risk stratification purposes [2, 4–6].

The Apgar score has demonstrated its utility in assessing the effectiveness of immediate resuscitation, as key components such as heart rate and respiratory effort should improve within 1, 5, and 10 or more minutes with successful measures [2]. It is also considered a reliable predictor of neonatal and infant mortality risk, particularly in preterm (< 34 weeks' gestation) and low birth weight infants (1500–2499 g) [7, 8].

Studies have shown an inverse relationship between 5-min Apgar scores and developmental vulnerability risk in the first 5 years, demonstrating the impact of other environmental and socioeconomic factors on child development [9].

Importantly, by 2022, the neonatal mortality rate in Ecuador was 5.4 per 1000 live births [10], nearly unchanged from the rate in 2017. Consequently, it highlights the urgent need to investigate the underlying health determinants affecting the neonatal population.

While prematurity and low birth weight remain primary predictors of neonatal mortality in low- and middle-income countries (LMICs) [11], including Ecuador, the Apgar score has also proven effective in forecasting early mortality among both non-at-risk and at-risk neonates [12]. Despite its longstanding use in diverse populations [7, 13–16], the predictive power of the Apgar score has yet to be evaluated in an Ecuadorian context. Based on the evidence discussed above, we hypothesize that a low Apgar score at 5 min could be directly and independently associated with early mortality (before 24 h) in Ecuadorian neonates.

## 2. Methods

### 2.1. Design

We conducted a retrospective case–control study of all neonates who were registered into the Surveillance System of Neonatal Mortality (SSNM) of the Ministry of Public Health of Ecuador, which only includes neonates deceased before the first 28 days of life, in a 3-year period from January 2014 to September 2017.

### 2.2. Database Context

According to the official data from the National Institute of Statistics and Censuses (INEC), which is a little different to the Ministry of Public Health of Ecuador data, the total population of Ecuador was estimated to be 16,726,458 in 2017; in the same year, there were 288,123 live births recorded; moreover, there was a neonatal mortality rate of 5.6 deaths per 1000 live births [17]. It is important to clarify that the calculation of neonatal mortality in Ecuador is based on data from INEC which is a little different from the mortality database employed in this study; this distinction is because, in Ecuador, many children are registered long after their birth, which can affect the accuracy and timeliness of the mortality data based only in data from the Ministry of Public Health of Ecuador.

**Figure 1:**
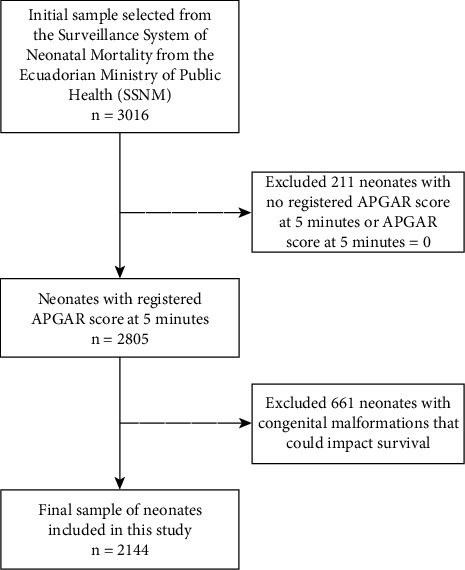
Flowchart diagram.

The time interval selected for this study, from January 2014 to September 2017, corresponds to the most recent data available provided by the Ministry of Public Health at the beginning of the analysis. The analysis of this data has extended to the present due to the duration of the analysis phase and manuscript preparation. Those neonates whose Apgar score is zero at 5 min and those with Apgar score at 5 min was not registered were excluded, as well as those neonates born with congenital malformations including hydranencephaly, congenital heart disease, diaphragmatic hernia, or polymalformation due to higher risk of early neonatal mortality [18, 19] (see the population flowchart diagram in Figure 1).

### 2.3. Patient and Public Involvement Statement

Patients or the public were not involved in the design, or conduct, or reporting, or dissemination plans of our research.

### 2.4. Population and Database

All registered neonatal deaths in public and private settings with available information on individual, contextual, and Apgar scores were included in this study. The explanation about the construction of the database is available in the supporting information.

### 2.5. Main Outcome

Cases were identified as neonates who died before 24 h of life, whereas controls were those neonates who survived beyond this period. We defined “early neonatal mortality” as death within the first 24 h, contrasting with the more classic definition of deaths within the first 0–7 days as used by UNICEF [20]. Our focus on the initial 24 h captures the highest risk period immediately after birth, ensuring accurate data collection within the healthcare setting. This definition is supported by other studies [21, 22], which also highlight the critical nature of this timeframe for addressing conditions like hypothermia, hypoglycemia, and birth asphyxia. Selection of controls was undertaken from the same registry, ensuring that they belonged to the same period and shared similar demographic characteristics, to minimize selection bias.

### 2.6. Main Explanatory Variable

Apgar score at 5 min was the main explanatory variable. We divided the score using a point-by-point *strata*, except in the case of scores ≤ 3 points. Stratifying Apgar scores point-by-point enhances neonatal health analysis precision, while grouping scores ≤ 3 addresses critical risks, simplifies data analysis, and focuses on urgent clinical intervention needs. We did not assess Apgar scores at 1 and 10 min due to the lack of data in the registry. However, recognizing the importance of effective neonatal resuscitation for low Apgar scores at 1 min and given that secondary and tertiary facilities typically have neonatal intensive care units with trained professionals, we included the level of healthcare facility as a covariate in our models to mitigate potential bias. We believe this has substantially reduced the impact of this limitation.

### 2.7. Other Covariates

Categorization of the Apgar score was executed using a point-by-point stratum, except for the score of ≤ 4 points. For the analysis, multilevel logistic regression models were used to estimate adjusted odds ratios (aORs) for neonatal death before 24 h, controlling for potential confounding variables. This approach allowed the comparison of the odds of neonatal mortality across different strata of the Apgar score.

The database provided access to a variety of covariates, categorized into two main groups: *(i)* individual covariates, including gestational age at birth, birth weight, classification of small for gestational age (< 10th percentile of birth weight for gestational age at birth) according to the Intergrowth equations [23], type of delivery, and comorbidities, and *(ii)* environmental covariates, which encompass the type and level of healthcare facility—Ecuadorian organization of healthcare is categorized into primary, secondary, and tertiary levels of care—as well as altitude. The altitude, measured in meters above sea level (m.a.s.l.), is categorized based on the methodology outlined in a previous study [24].

### 2.8. Statistical Analyses and Sample Size Considerations

The analyses were performed using the entire Ecuadorian neonatal mortality data registry, which began in 2014 and had information only until 2017 at the time of data collection. Prior to conducting the analyses, we calculated the sample size needed to achieve statistical power. The calculation was based on a 1:1 matched-pair design with the objective of achieving an 80% power at a 5% significance level. The estimated probability of exposure in the control group was less than 10%. To detect an odds ratio (OR) of 10 with adequate statistical power, a total of 171 cases were required. According to the sample size calculation, we had sufficient number of neonates in case and control to discern statistically significant differences in odds of the exposure according to the outcome [25].

Descriptive statistics were performed using percentages for categorical variables and the median, lower quartile, and upper quartile for discrete variables. To assess the differences of the clinical and environmental variables between neonates who died < 24 and ≥ 24 h of life, we performed *(i)* Kruskal–Wallis tests for assessing differences of gestational age (given this is a nonnormal distributed variable), *(ii)t*-test for assessing differences in birth weight (given it was a normally distributed variable), and *(iii)* chi^2^ tests for assessing differences in variables of small for gestational age, type of delivery, comorbidities, type of healthcare facility, and level of care.

Then, we examined the bivariate relationships between individual covariates (gestational age at birth, birth weight, classification of small for gestational age (< 10th percentile of birth weight for gestational age at birth) according to the Intergrowth equations, type of delivery, and comorbidities) and environmental covariates, which encompass the type and level of healthcare facility and early death by multilevel logistic regression. The random effects variable in this model was altitude at which neonates were attended; this component of the model allows the intercept estimates to vary randomly among the groups of altitude, suggesting that there are differences between these groups that could influence the study results at which the neonates were attended.

Subsequently, multilevel logistic regression models were used to estimate aORs for neonatal death before 24 h, controlling for potential confounding variables. We considered a list of potential confounding factors based on clinical expertise of the researchers. These included individual and contextual variables. Using these factors, we constructed a “saturated model” that included factors independently associated with either the exposure or the outcome (*p* value < 0.05) or that significantly altered the estimates for other variables. We then refined this model to a “parsimonious model” through an approached based on backward stepwise elimination process, removing factors one by one based on Wald's *p* value > 0.15, and according to the clinical expertise of researchers. Collinearity between variables specially between birth weight and gestational age was assessed in both the saturated and parsimonious models using the variance inflation factor (VIF). As standard VIF diagnostics are not available for logistic regression, linear regression models were employed to compute VIF values.

We performed several secondary analyses to assess the sensitivity of our estimates to our assumptions regarding potential biases, as well as to test for model misspecification. Considering that differential treatment for individual causes of death could affect the estimates, we ran the model excluding neonates who died from *(i)* asphyxia-related disorders, *(ii)* congenital malformations, *(iii)* prematurity-related disorders, *(iv)* infectious disorders, and *(v)* other diseases that were not classified in the previous referred groups. Given the small number of missing data (< 6%, see footnotes in Table 1), we employed a complete case analysis in estimating statistical associations. We considered that there were statistically significant differences when the *p* value < 0.05. Finally, analyses were performed with Stata 14.2 (*Statistical Software Stata: Release 14.2 College Station, Texas: StataCorp LP*).

### 2.9. Ethical Issues

This study was part of the “Score Bebé” project and conducted with the approval of the Research Ethics Committee in Human Beings (*CEISH*) of the *Pontificia Universidad Católica del Ecuador* (code number 2018-09-EO). An earlier version of this work was previously made available as a preprint on MedRxiv [26]. This manuscript has undergone substantial revisions and expansions from its preprint version, prompted by significant changes suggested during a previous submission to another journal, which necessitated a complete restructuring of this paper. Modifications include changes to the title, the objectives in the introductory section, the design, the health outcome variable, the descriptive and statistical analyses, the construction of model types, and the models themselves. There have been extensive revisions throughout the discussion section, most of the bibliographic references, and the conclusions. These alterations have significantly deepened and broadened the scope of our analysis, yielding new insights and results that were not present in the original preprint.

## 3. Results

A total of 2144 deceased neonates were included, with a slight majority being male (53.2%). Early neonatal death (<24 h) occurred in 581 (27.1%) neonates of the total. The mean gestational age at birth was approximately 30.8 weeks, with a significant portion of the neonates (29.9%) born at less than 28 weeks. The mean birth weight was reported as 1525.2 g, with 18.0% of neonates weighing less than 750 g at birth. A considerable number of deliveries were performed via caesarean section (56.0%). Regarding comorbidities, prematurity was the most prevalent (39.4%), followed by asphyxia (31.2%) and infections (24.7%). The distribution of Apgar scores revealed a notable pattern of neonatal health outcomes; specifically, 17.4% (*n* = 374) were categorized with Apgar scores of 3 or below, 16.1% (*n* = 345) of neonates had a score of 7, 15.9% (*n* = 341) had a score of 8, 16.5% (*n* = 354) with a score of 9, and only 47 (2.2%) had a score of 10. Most neonates were born in public healthcare facilities (74.0%) and resided in urban areas (72.2%) (see Table 1).

Statistically significant differences were observed in gestational age between the two groups, notably a higher percentage of neonates born at less than 28 weeks' gestation among those who died before 24 h (45.8% compared to 24.1%); additionally, a greater percentage of neonates born at more than 41 weeks' gestation were among those who died before 24 h (3.3% vs. 1.7%). Regarding birth weight, there were also significant differences between the two groups, with a higher percentage of neonates weighing less than 750 g at birth among those who died before 24 h compared to those who did not (30.6% vs. 13.4%). The mode of delivery also differed between the groups; specifically, there was a higher percentage of cephalic eutocic vaginal deliveries and dystocic deliveries among those who died before and after 24 h (43.1% compared to 36.0% and 9.5% compared to 4.5%, respectively). As for the differences in the 5-min Apgar score, there were differences in the percentages of neonates with each score between those who died before and after 24 h; specifically, there was a higher frequency of neonates with an Apgar score of 3 or less at 5 min compared to their counterparts (41.7% vs. 8.5%) and a lower percentage of neonates with scores of 9 and 10 (8.1% vs. 19.6% and 0.5% vs. 2.8%, respectively). A nonsignificant difference (*p* value = 0.082) was observed in the distribution of neonatal deaths across altitude categories; in that regard, higher proportion of neonates who died within 24 h were from areas at ≥ 2750 m (27.7% vs. 26.2%), while a lower proportion were from < 80 m (53.4% vs. 55.7%). The comparison of the characteristics of neonates who died before (581) and ≥ 24 h (1581) of life is provided (see Table 2).

According to the final (parsimonious) model, neonates born after 28 weeks exhibit a lower association with mortality before 24 h compared to those born before 28 weeks, notably the group from > 28 to 31 weeks with an aOR of 0.53 (95% CI: 0.37–0.74, *p* < 0.001) and the group from >38 to <40 weeks with an aOR of 0.33 (95% CI: 0.16–0.69, *p* = 0.003), using those born before 28 weeks of gestational age as the reference group. Regarding birth weight, neonates weighing between 750 and 1000 g at birth show a lesser association with early mortality than those under 750 g, with an OR of 0.62 (95% CI: 0.43–0.87, *p* = 0.06); this association decreases in neonates with higher weight ranges. As for the type of delivery, there is a higher association of early neonatal mortality with cephalic eutocic vaginal and dystocic births compared to caesarean sections, with ORs of 1.34 (95% CI: 1.06–1.69, *p* = 0.016) and 1.79 (95% CI: 1.13–2.84, *p* = 0.013), respectively. In terms of comorbidities, neonatal infections show a lower association with early mortality compared to disorders related to asphyxia, with an OR of 0.32 (95% CI: 0.23–0.46, *p* < 0.001). Finally, the 5-min Apgar score demonstrates a strong association with neonatal mortality before 24 h. An Apgar score ≤ 3 is significantly associated with increased early mortality aOR of 20.65 (95% CI: 5.99–71.28, *p* < 0.001), a trend that persists as the Apgar score decreases. The aORs for neonatal death before 24 h showing an inverse association between gestational age at birth and early neonatal mortality can be seen (see Table 3). Birth weight and gestational age were evaluated for collinearity using the VIF, with the highest values being 5.66 and 3.72, respectively, both below the threshold of 10, indicating no severe collinearity. In the final parsimonious model, the mean VIF remained low (4.39), and no variable exceeded the critical threshold of 10, confirming that collinearity did not significantly affect model performance.

Sensitivity analyses were performed to assess if the estimates changed with specific exclusions yielding comparable results too (see Table S1). Consequently, excluding *(i)* asphyxia-related disorders, *(ii)* congenital malformations, *(iii)* prematurity-related disorders, *(iv)* infectious disorders, and *(v)* those neonates who were not singleton did not change the estimates.

## 4. Discussion

A clear and robust association exists between the 5-min Apgar score and early neonatal mortality (before 24 h) in Ecuadorian neonates. This relationship persists even after adjusting for factors like mode of delivery, comorbidities, asphyxia, prematurity, infections, and other complications, as well as varying care levels from primary to tertiary. Consequently, the Apgar score proves to be a crucial predictor, highlighting its vital role in clinically assessing neonates and helping healthcare practitioners identify those at higher risk.

We have determined a robust and inversely proportional relationship between the 5-min Apgar score and the estimation of neonatal early death risk. Numerous studies have shown that the Apgar scale is associated with not only neonatal mortality but also later mortality [5, 7, 14, 15, 27, 28]. Our study focused solely on neonates who died; an Apgar score of ≤ 3 reported an OR of 21 for early neonatal death, with confidence intervals reaching up to 134 in crude models and 71 in adjusted models. This contrasts with findings from broader cohort studies, such as one reporting a hazard ratio (HR) of 961.7 (95% CI: 681.3–1357.5), which includes both survivors and nonsurvivors [7]. While the OR of 21 reflects the association within a sample of deceased neonates, the HR of 961.7 in large cohorts provides a more precise estimate of the risk of neonatal death as a function of time and in a more representative population. The absence of a direct comparison group within our study limits statistical analysis and interpretation, potentially affecting the magnitude of associations due to the potential selection bias of a case–control design. Ideally, a comparison with neonates who survived would provide a more comprehensive assessment of neonatal mortality risks.

These findings indicate that the neonate's reaction to the transition of life is pivotal for subsequent health outcomes, such as neonatal morbidity [16, 29] and mortality [6]. Our findings must be interpreted under the fact that it was conducted among a vulnerable population, considering that the entirety of the sample passed away during the neonatal period. This could imply a heightened risk, adverse social, accessibility, and clinical determinants during pregnancy and childbirth, compared to the general population of Ecuador. Notably, most deceased neonates in our study were attended in public healthcare facilities. While this may reflect the broader coverage of the public health system, it also underscores the need to assess factors influencing neonatal care in these settings, recognizing that early neonatal mortality is shaped not only by biomedical factors but also by social and environmental determinants [30].

It is also important to consider that nonclinical factors, such as limited prenatal care [31] and challenges in timely referrals and neonatal transportation [32], may also play a role in neonatal outcomes in Ecuador. Additionally, limited staff training in neonatal resuscitation and resource constraints may contribute to the centralization of neonatal care in tertiary hospitals, potentially impacting neonatal outcomes in Ecuador. It is important to mention that neonatal mortality in Ecuador remains a significant public health challenge, highlighting the need for continued evaluation and improvement of neonatal care [33]. Importantly, and as part of the nonclinical factor analysis, we identified an association between higher altitudes and early neonatal mortality, aligning with findings from a previous study. However, a detailed evaluation of altitude's impact falls beyond the scope of this study. For a more comprehensive analysis, including potential physiological and healthcare access mechanisms, please refer to our previous work [24].

Interestingly, when both gestational age categories and birth weight were included in the model, a reduction in the statistical significance of the birth weight variable was observed. This suggests that gestational age is a little more robust determinant of neonatal mortality compared to weight at birth but it seems important to include both variables [34, 35]. Despite the fact that there is evidence that demonstrated the fundamental importance of gestational age versus birth weight [36], other recent studies support the importance of both, gestational age and birth weight, as mortality predictors in neonatal period [34]; we consider it is necessary to have more research to elucidate the strength of each variable on predicting neonatal outcomes [37].

We found that vaginal dystocic delivery, including cephalic eutocic vaginal delivery, is a factor associated with higher odds of early neonatal mortality compared to caesarean delivery. In our study, caesarean delivery was associated with lower early neonatal mortality, consistent with findings from other studies in Latin America and developing countries [38], but its rationale is not frequently warranted, maybe because of a lack of humanized care that prioritizes the autonomy, dignity, and cultural preferences of the birthing person, ensuring family support, minimizing unnecessary medical interventions, and advocating for evidence-based practices during birth attendance [38] or by the idea that the patient has the right to choose a delivery method, even, in the absence of an indication for a caesarean [39]; therefore, access to this intervention should be carefully evaluated, with the potential involvement of local quality control departments in health facilities to ensure its appropriate use [40]. Additionally, promoting evidence-based decision-making, particularly for at-risk pregnant women, may contribute to more effective and equitable care [41].

Since our study sample was entirely composed of deceased neonates, we consider it to be a group of patients with a baseline risk condition that let us to consider them as “at-risk” population. Our findings emphasize the importance of timely and comprehensive obstetric care in reducing early neonatal mortality. Institutional childbirth care by trained professionals and adequate antenatal care remain essential in improving neonatal outcomes and mitigating risks [42–45].

Our findings indicate that neonates attended in secondary and tertiary care facilities had lower estimated odds of mortality compared to those in primary care settings. This could be related to the previously mentioned aspects regarding the comprehensiveness of care and the access of at-risk pregnant women to such care and, specially, the access to a caesarean section, which is provided only in secondary and tertiary care, when necessary [46, 47]. Specifically, it may indicate the need to strengthen both primary and secondary levels of obstetric and perinatal care to reduce neonatal mortality and morbidity rates. Scientific evidence asserts similar claims [48].

Among the strengths of our study, it is important to highlight the use of a large database with national coverage. This data was derived from neonatal mortality of the Ministry of Public Health and, therefore, reflects real-world scenarios in the Ecuadorian context, which is not very common in studies conducted at regional level due to the significant difficulty in accessing similar databases, especially prospective databases from broader cohorts that allow comparison between those who survive and those who do not. The inclusion of maternal, neonatal, and contextual clinical variables enables us to adjust our associations for these variables, avoiding multicollinearity or even overfitting. This is because we have rigorously selected the variables, employing the clinical judgment of pediatricians and neonatologists, as well as other doctors and health professionals on our research team.

Among the limitations of our study, it is crucial to highlight that the entirety of Ecuadorian mortality data from 2014 to 2017, which could be recorded in the neonatal mortality surveillance database of the Ministry of Public Health and considering these were neonates who died in their entirety, did not allow for a survival analysis. Such analysis could have enabled a more objective and accurate examination of the determinants of neonatal mortality in the Ecuadorian population. The absence of a comparative group of neonates who survived poses a constraint. Consequently, the main limitation of our study was inherent to its case–control design, namely, the inability to establish risk associations but rather ORs that facilitate the formulation of hypotheses for future cohort studies.

Another potential limitation is the subjective assessment of the Apgar score; specifically, the Ecuadorian health system has medical interns and rural doctors, who often are the ones determining the Apgar score; consequently, the values depend greatly on the operator. It could diminish the precision of our findings. However, the differences in the Apgar score are statistically significant concerning the risk estimation of neonatal death. This suggests that while the precision in evaluating the Apgar score could be considered a limitation, it is noteworthy that when conducted in a real-world setting, the Apgar score shows to be a good predictor of early neonatal death. This assertion withstands the sensitivity analyses we have applied. A significant limitation of our study is the potential variability in the implementation of neonatal resuscitation protocols [49] and the Apgar scoring system across hospitals. Although national guidelines mandate standardized training and use of these protocols [50, 51], the extent to which they were effectively socialized and consistently applied is uncertain. This variability could influence the accuracy of Apgar scores and the effectiveness of neonatal resuscitation, which may affect our study's findings.

## 5. Conclusions

This study demonstrated that the Apgar score, among other determinants, was significantly associated with early neonatal mortality (before the first 24 h). This association was independent of type of delivery, comorbidities, disorders related to asphyxia, prematurity, infections, other medical disorders, as well as varying levels of care from primary to tertiary. These findings underscore the importance of Apgar evaluation in neonates and suggest a predictive value of the score for early neonatal mortality.

## Figures and Tables

**Table 1:** Characteristics of neonates who were included in the study and were registered by the Surveillance System of Neonatal Mortality (SSNM) of the Ministry of Public Health of Ecuador.

**Variables**	**Total sample** ^**a**^ ** (** **n** ** = 2144)** **Frequency, mean, or median**
Gender	
Male	1141 (53.22%)
Female	1003 (46.78%)
Gestational age (weeks)	Mean (SD): 30.82 (5.06)
< 28 weeks	642 (29.94%)
> 27–31 weeks	599 (27.94%)
> 31–36 weeks	496 (23.13%)
> 36–38 weeks	208 (9.70%)
> 38–< 41 weeks	153 (7.14%)
> 40 weeks	46 (2.15%)
Birth weight (g)	Mean (SD): 1525.22 (892.07)
<750 g at birth	385 (18.02%)
750–< 1000 g at birth	414 (19.38%)
1000–< 1500 g at birth	507 (23.74%)
1500–< 2500 g at birth	452 (21.16%)
2500–< 4000 g at birth	361 (16.90%)
4000 g or more at birth	17 (0.80%)
Birth weight percentile for gestational age	
5th–95th percentile	1521 (74.82%)
< 5th percentile	397 (19.53%)
> 95th percentile	115 (5.66%)
Type of delivery	
Caesarean	1189 (55.98%)
Eutocic vaginal delivery	810 (38.14%)
Dystocic delivery	125 (5.89%)
Comorbidities^b^	
Asphyxia	668 (31.16%)
Prematurity	845 (39.41%)
Infections	530 (24.72%)
Other	101 (4.71%)
Apgar score at 5 min, median (P25–P75)	Median (P25–P75): 4 (3–6)
≤ 3, *n* (%)	374 (17.44%)
4, *n* (%)	136 (6.34%)
5, *n* (%)	243 (11.33%)
6, *n* (%)	304 (14.18%)
7, *n* (%)	345 (16.09%)
8, *n* (%)	341 (15.90%)
9, *n* (%)	354 (16.51%)
10, *n* (%)	47 (2.19%)
Prenatal care	
Received	1723 (80.36%)
Not received	421 (19.64%)
Level of care	
Primary	40 (1.87%)
Secondary	1015 (47.56%)
Tertiary	1079 (50.56%)
Locality	
Rural	596 (27.80%)
Urban	1548 (72.20%)
Type of facility	
Private	557 (25.98%)
Public	1587 (74.02%)
Altitude (m)	
< 80	1180 (55.04%)
≥ 80–< 2500	284 (13.25%)
2500–< 2750	109 (5.08%)
≥ 2750	571 (26.63%)
Death before 24 h	
No	1563 (72.90%)
Yes	581 (27.10%)

*Note:* Altitude is expressed in meters above sea level. Intergrowth categories: based on the Intergrowth-21st Project for assessing fetal and neonate growth.

^a^The dataset comprehensively includes 2144 neonatal records, with minimal missing data across several key variables. Gestational age is available for 2135 neonates, with 9 records missing (0.42%). Birth weight is reported for 2136 neonates, with 8 records missing (0.37%). Birth weight percentile for gestational age has 2104 valid entries, reflecting 40 missing data points (1.87%). Type of delivery information is available for 2124 neonates, leaving 20 records missing (0.93%). Level of care is recorded for 2134 cases, with 10 missing values (0.47%).

^b^Comorbidities are classified according to the corresponding ICD-10 codes, as detailed in Table S1.

**Table 2:** Comparison of neonatal mortality before and after 24 h by various characteristics.

**Variable/category**	**Deat ≥ 24 h (** **n** ** = 1563)**	**Death < 24 h (** **n** ** = 581)**	**p** ** value**
Neonate sex			
Male, *n* (%)	844 (54.00%)	297 (51.12%)	0.235
Female, *n* (%)	719 (46.00%)	284 (48.88%)	
Gestational age in weeks, median (P25–P75)	31 (28–35)	28 (25–34)	< 0.001
Birth weight in grams, mean (standard deviation)	1557.4 (865.0)	1438.7 (956.5)	0.006
Intergrowth categories			
5th–95th percentile, *n* (%)	1110 (75.20%)	411 (73.79%)	0.146
< 5th percentile, *n* (%)	276 (18.70%)	121 (21.72%)	
> 95th percentile, *n* (%)	90 (6.10%)	25 (4.49%)	
Type of delivery			
Caesarean, *n* (%)	915 (58.79%)	274 (47.40%)	< 0.001
Eutocic cephalic vaginal birth, *n* (%)	561 (36.03%)	249 (43.08%)	
Dystocic birth, *n* (%)	70 (4.49%)	55 (9.52%)	
Comorbidities			
Asphyxia-related disorders, *n* (%)	437 (28.06%)	231 (39.76%)	< 0.001
Prematurity-related disorders, *n* (%)	574 (36.87%)	271 (46.64%)	
Infection-related disorders, *n* (%)	475 (30.51%)	55 (9.47%)	
Other disorders, *n* (%)	77 (4.95%)	24 (4.13%)	
Apgar score at 5 min, median (P25–P75)	7 (5–8)	5 (2–7)	< 0.001
Prenatal care			
No prenatal control, *n* (%)	302 (19.32%)	119 (20.48%)	0.548
One or more prenatal controls, *n* (%)	1261 (80.68%)	462 (79.52%)	
Level of care			
Primary level, *n* (%)	22 (1.41%)	18 (3.12%)	0.003
Secondary level, *n* (%)	721 (46.31%)	294 (50.95%)	
Tertiary level, *n* (%)	814 (52.28%)	265 (45.93%)	
Locality			
Rural, *n* (%)	434 (27.77%)	162 (27.88%)	0.958
Urban, *n* (%)	1129 (72.23%)	419 (72.12%)	
Type of facility			
Private, *n* (%)	418 (26.74%)	139 (23.92%)	0.186
Public, *n* (%)	1145 (73.26%)	442 (76.08%)	
Altitude (m)			
< 80, *n* (%)	870 (55.66%)	310 (53.36%)	0.082
≥ 80–< 2500, *n* (%)	214 (13.69%)	70 (12.05%)	
2500–< 2750, *n* (%)	69 (4.41%)	40 (6.88%)	
≥ 2750, *n* (%)	410 (26.23%)	161 (27.71%)	

*Note:* Comorbidities are classified according to the corresponding ICD-10 codes, as detailed in Table S1. Altitude is expressed in meters above sea level. Intergrowth Categories: based on the Intergrowth-21st Project for assessing fetal and neonate growth.

**Table 3:** Multilevel logistic regression: Crude and adjusted (from saturated and parsimonious models) odds ratios for neonatal mortality before 24 h.

**Variable/category**	**Crude odds ratio (95% CI)**	**p** ** value**	**Adjusted odds ratio (95% CI)** **Saturated model**	**p** ** value**	**Adjusted odds ratio (95% CI)** **Parsimonious model**	**p** ** value**
Male (*female is the ref.*)	1.12 (0.93–1.36)	0.235	1.02 (0.81–1.27)	0.891	—	—
Gestational age in weeks						
< 28 weeks (*ref.*)	1	—	1	—	1	—
≥ 28–31 weeks	0.33 (0.25–0.43)	< 0.001	0.56 (0.39–0.79)	0.001	0.53 (0.37–0.74)	< 0.001
> 31–36 weeks	0.34 (0.26–0.45)	< 0.001	0.49 (0.30–0.79)	0.004	0.46 (0.29–0.75)	0.002
> 36–38 weeks	0.39 (0.27–0.56)	< 0.001	0.53 (0.27–1.04)	0.066	0.46 (0.24–0.90)	0.023
> 38–< 40 weeks	0.52 (0.35–0.76)	0.001	0.39 (0.18–0.82)	0.013	0.33 (0.16–0.69)	0.003
> 41 weeks	0.99 (0.54–1.83)	0.986	0.85 (0.33–2.22)	0.740	0.74 (0.29–1.88)	0.525
Birth weight in grams, *mean* (*standard deviation*)						
< 750 g at birth (*ref.*)	1	—	1	—	1	—
750–< 1000 g	0.36 (0.27–0.49)	< 0.001	0.62 (0.43–0.88)	0.008	0.62 (0.43–0.87)	0.006
1000–< 1500 g	0.30 (0.22–0.41)	< 0.001	0.76 (0.51–1.16)	0.203	0.82 (0.55–1.23)	0.346
1500–< 2500 g	0.30 (0.22–0.41)	< 0.001	1.04 (0.61–1.75)	0.897	1.15 (0.69–1.91)	0.598
2500–< 4000 g	0.47 (0.35–0.64)	< 0.001	1.40 (0.71–2.74)	0.328	1.69 (0.88–3.26)	0.117
4000 g or more	0.36 (0.12–1.14)	0.080	0.35 (0.07–1.65)	0.183	0.47 (0.10–2.16)	0.329
Intergrowth categories						
5th–95th percentile (*ref.*)	1	—	1	—	—	
< 5th percentile	1.18 (0.93–1.51)	0.171	1.20 (0.91–1.59)	0.201	—	—
> 95th percentile	0.75 (0.47–1.19)	0.218	0.78 (0.46–1.30)	0.341	—	—
Type of delivery						
Caesarean (*ref.*)	1	—	1	—	1	—
Eutocic cephalic vaginal birth	1.48 (1.21–1.81)	< 0.001	1.32 (1.03–1.69)	0.026	1.34 (1.06–1.69)	0.016
Dystocic birth	2.62 (1.80–3.83)	< 0.001	1.96 (1.22–3.17)	0.006	1.79 (1.13–2.84)	0.013
Comorbidities^a^						
Asphyxia-related disorders	1	—	1	—	1	—
Prematurity-related disorders	0.89 (0.72–1.11)	0.303	0.86 (0.65–1.12)	0.263	0.87 (0.67–1.13)	0.301
Infection-related disorders	0.22 (0.16–0.30)	< 0.001	0.31 (0.22–0.45)	< 0.001	0.32 (0.23–0.46)	< 0.001
Other disorders	0.59 (0.36–0.96)	0.033	0.75 (0.42–1.33)	0.318	0.73 (0.42–1.26)	0.263
Apgar score at 5 min						
10 (*ref.*)	1	—	1	—	1	—
9	2.07 (0.61–6.97)	0.240	3.37 (0.76–14.89)	0.109	2.16 (0.62–7.49)	0.226
8	2.84 (0.85–9.53)	0.090	3.74 (0.84–16.56)	0.083	2.61 (0.75–9.08)	0.131
7	3.02 (0.90–10.12)	0.074	3.73 (0.84–16.51)	0.083	2.49 (0.71–8.67)	0.152
6	4.57 (1.36–15.32)	0.014	5.60 (1.26–24.89)	0.024	3.75 (1.07–13.12)	0.039
5	6.96 (2.08–23.31)	0.002	7.18 (1.62–31.81)	0.009	4.90 (1.41–17.07)	0.013
4	7.49 (2.18–25.73)	0.001	8.78 (1.94–39.73)	0.005	5.45 (1.52–19.48)	0.009
≤ 3	30.18 (9.12–99.82)	< 0.001	30.54 (6.95–134.23)	< 0.001	20.65 (5.99–71.28)	< 0.001
No prenatal control	1.08 (0.85–1.37)	0.548	0.97 (0.70–1.35)	0.853	—	—
Rural locality (urban is the ref.)	0.99 (0.80–1.22)	0.958	1.05 (0.81–1.37)	0.687	—	—
Level of care					—	—
Primary level (*ref.*)	1	—	1	—	1	—
Secondary level	0.50 (0.26–0.94)	0.032	0.64 (0.29–1.41)	0.268	0.67 (0.31–1.43)	0.302
Tertiary level	0.39 (0.21–0.75)	0.005	0.48 (0.22–1.06)	0.070	0.46 (0.21–0.99)	0.048
Public institution (*private is the ref.*)	1.16 (0.93–1.45)	0.186	1.27 (0.89–1.80)	0.193	—	—

^a^Comorbidities are classified according to the corresponding ICD-10 codes, as detailed in Table S1. Intergrowth categories: based on the Intergrowth-21st Project for assessing fetal and neonate growth.

## Data Availability

The data that support the findings of this study are available from the corresponding author upon reasonable request.

## Supporting Information

Supporting Information Additional supporting information can be found online in the Supporting Information section. The supporting information provides a detailed overview of the methodology and protocols involved in building a database for neonatal mortality data, lists and describes the roles and responsibilities of health system actors obligated to report neonatal deaths, discusses the established criteria for reporting these deaths to ensure data consistency and accuracy, and outlines the verification procedures used to ensure the reliability and validity of the reported information. Figure S1: Histogram of the distribution of neonatal deaths per Apgar scale score at 5 min (page 3 of the supporting information)—this histogram provides a visual representation of the distribution of neonatal deaths according to the Apgar score at 5 min. See Figure S1 in the supporting information for detailed distribution analysis. Table S1: Multilevel logistic regression: crude and adjusted odds ratios for neonatal mortality before 24 h (page 4 of the supporting information)—this table presents the results of a multilevel logistic regression analysis, providing both crude and adjusted odds ratios for neonatal mortality before 24 h, excluding asphyxia-related disorders, prematurity-related disorders, infectious disorders, and nonsingleton cases. Refer to Table S2 in the supporting information for a comprehensive view of the statistical outcomes.
