# Supplementary material for: Early Neonatal Mortality (< 24 h) in Ecuador: A Population-Based Study on the Impact of Apgar Score, Gestational Age, Birth Weight, Delivery Type, and Healthcare Level
Source: Int J Pediatr. 2025 May 19;2025:4225987. doi: 10.1155/ijpe/4225987 (PMC12105887; doi:10.1155/ijpe/4225987)
Supplement: Supporting Information — Additional supporting information can be found online in the Supporting Information section. The supporting information provides a detailed overview of the methodology and protocols involved in building a database for neonatal mortality data, lists and describes the roles and responsibilities of health system actors obligated to report neonatal deaths, discusses the established criteria for reporting these deaths to ensure data consistency and accuracy, and outlines the verification procedures used to ensure the reliability and validity of the reported information. Figure S1: Histogram of the distribution of neonatal deaths per Apgar scale score at 5 min (page 3 of the supporting information)—this histogram provides a visual representation of the distribution of neonatal deaths according to the Apgar score at 5 min. See Figure S1 in the supporting information for detailed distribution analysis. Table S1: Multilevel logistic regression: crude and adjusted odds ratios for neonatal mortality before 24 h (page 4 of the supporting information)—this table presents the results of a multilevel logistic regression analysis, providing both crude and adjusted odds ratios for neonatal mortality before 24 h, excluding asphyxia-related disorders, prematurity-related disorders, infectious disorders, and nonsingleton cases. Refer to Table S2 in the supporting information for a comprehensive view of the statistical outcomes. [file 4225987.f1.docx]

**Early Neonatal Mortality in Ecuador: A Population-Based Study on the Impact of Apgar Score, Gestational Age, Birth Weight, Delivery Type, and Health Care Level**

(ONLINE SUPPLEMENTARY MATERIAL)

Dueñas-Espín, I. *et al*.

[Process of building the database 2](#_Toc190161184)

[Actors of the health system which are obligated to report neonatal deaths 2](#_Toc190161185)

[Established criteria for reporting neonatal deaths 2](#_Toc190161186)

[Verifying information 2](#_Toc190161187)

[Figure 1S.- Histogram of the distribution of neonatal deaths per Apgar scale score at 5 minutes. 3](#_Toc190161188)

[Table 1S: Neonatal comorbidities and corresponding ICD-10 codes. 4](#_Toc190161189)

[Table 2S: Multilevel Logistic Regression: Crude and Adjusted Odds Ratios for Neonatal Mortality Before 24 Hours excluding: (i) asphyxia related disorders, (ii) prematurity related disorders, (iii) infectious disorders, and (iv) those who were not singleton. 5](#_Toc190161190)

## Process of building the database

The database was built based on the information collected from all hospitals in the Public Network of Health Establishments (made up of all public hospitals and its associates) and the Private Network of Health Establishments, including all Specialty Hospitals of Gynaecology and Obstetrics, as well as any hospital that attends births. The database is built from systematic reports from the health establishments whenever there is a newborn death (≤28 days of life). The reports are sent in through a pre-established form by using a unique username and password for each establishment. This username is assigned by the system manager (DNVE).

### Actors of the health system which are obligated to report neonatal deaths

All public and private health establishments in the country are obligated to report any neonatal death in the first 24 hours after the event.

### Established criteria for reporting neonatal deaths

There are two pre-established forms and two ways to notify neonatal deaths in the system. The first one is a simplified report where all neonatal deaths are reported. The second one registers both the information from the simplified report and other additional information, including criteria that show preventability. This data includes some anonymized information from the mother.

### Verifying information

There is an epidemiologist responsible for each political-administrative zone. This epidemiologist has a username and password that gives him/her access to all the information reported in the designated area. The epidemiologist is responsible for reviewing the information and detecting incongruences, by verifying with those who report it.

## Figure 1S.- Histogram of the distribution of neonatal deaths per Apgar scale score at 5 minutes.

## Table 1S: Neonatal comorbidities and corresponding ICD-10 codes.

| **Category** | **ICD-10 Code Examples** | **Description** |
| --- | --- | --- |
| **Asphyxia-related disorders** | P21.0, P91, P10.2 | Severe birth asphyxia, other brain damage of neonate, intraventricular haemorrhage due to birth injury |
| **Prematurity-related disorders** | P07, P07.2, P07.3 | Disorders related to short gestation and low birth weight, extreme immaturity, other preterm neonates |
| **Infection-related disorders** | P36, P36.9, Q89 | Bacterial sepsis of neonate, unspecified bacterial sepsis, congenital malformations potentially associated with infection-related complications |
| **Other disorders** | P03, P77, P22, P22.0, R57.0 | Complications of labour and delivery, necrotising enterocolitis, neonatal respiratory distress syndrome, cardiogenic shock |

## Table 2S: Multilevel Logistic Regression: Crude and Adjusted Odds Ratios for Neonatal Mortality Before 24 Hours excluding: (i) asphyxia related disorders, (ii) prematurity related disorders, (iii) infectious disorders, and (iv) those who were not singleton.

|  | **Excluding neonates with asphyxia related disorders** | | **Excluding Prematurity-Related Disorders** | | **Excluding Infection-Related Disorders** | | **Excluding neonates who were not singleton** | |
| --- | --- | --- | --- | --- | --- | --- | --- | --- |
| **Variable / Category^*^** | **Odds Ratio (95% CI)** | **p-value** | **Odds Ratio (95% CI)** | **p-value** | **Odds Ratio (95% CI)** | **p-value** | **Odds Ratio (95% CI)** | **p-value** |
| **Gestational Age in weeks** |  |  |  |  |  |  |  |  |
| < 28 weeks (ref.) | 1 | - | 1 | - | 1 | - | 1 | - |
| ≥28 to 31 weeks | 0.56 (0.37 - 0.86) | 0.008 | 0.44 (0.27 - 0.72) | 0.001 | 0.54 (0.37 - 0.78) | 0.001 | 0.55 (0.39 - 0.78) | 0.001 |
| >31 to 36 weeks | 0.51 (0.28 - 0.93) | 0.027 | 0.37 (0.19 - 0.72) | 0.003 | 0.45 (0.27 - 0.75) | 0.002 | 0.48 (0.30 - 0.77) | 0.002 |
| >36 to 38 weeks | 0.50 (0.20 - 1.25) | 0.140 | 0.39 (0.17 - 0.88) | 0.023 | 0.45 (0.22 - 0.95) | 0.036 | 0.47 (0.24 - 0.92) | 0.028 |
| >38 to <40 weeks | 0.33 (0.10 - 1.07) | 0.064 | 0.30 (0.13 - 0.70) | 0.006 | 0.35 (0.16 - 0.77) | 0.009 | 0.35 (0.17 - 0.72) | 0.004 |
| >41 weeks | 0.36 (0.09 - 1.52) | 0.166 | 0.58 (0.20 - 1.70) | 0.319 | 1.12 (0.38 - 3.29) | 0.836 | 0.77 (0.30 - 1.96) | 0.577 |
| **Birth Weight in g, mean (standard deviation)** |  |  |  |  |  |  |  |  |
| < 750g at birth (ref.) | 1 | - | 1 | - | 1 | - | 1 | - |
| 750 to <1000g | 0.63 (0.41 - 0.95) | 0.029 | 0.88 (0.52 - 1.50) | 0.647 | 0.53 (0.37 - 0.78) | 0.001 | 0.62 (0.44 - 0.88) | 0.008 |
| 1000 to <1500g | 0.72 (0.43 - 1.20) | 0.204 | 1.10 (0.61 - 1.96) | 0.753 | 0.85 (0.55 - 1.31) | 0.463 | 0.83 (0.55 - 1.24) | 0.364 |
| 1500 to <2500g | 1.02 (0.54 - 1.93) | 0.945 | 1.41 (0.68 - 2.91) | 0.358 | 1.21 (0.70 - 2.07) | 0.495 | 1.16 (0.69 - 1.92) | 0.579 |
| 2500 to <4000g | 1.81 (0.74 - 4.45) | 0.197 | 2.29 (1.00 - 5.24) | 0.050 | 1.57 (0.77 - 3.21) | 0.216 | 1.70 (0.88 - 3.28) | 0.114 |
| 4000g or more | - | - | 0.70 (0.14 - 3.49) | 0.662 | 0.49 (0.10 - 2.39) | 0.379 | 0.47 (0.10 - 2.19) | 0.338 |
| **Type of Delivery** |  |  |  |  |  |  |  |  |
| Cesarean (ref.) | 1 | - | 1 | - | 1 | - | 1 | - |
| Eutocic Cephalic Vaginal Birth | 1.51 (1.12 - 2.05) | 0.007 | 1.19 (0.87 - 1.64) | 0.275 | 1.28 (0.99 - 1.65) | 0.062 | 1.33 (1.05 - 1.68) | 0.020 |
| Dystocic Birth | 2.19 (1.15 - 4.18) | 0.018 | 1.52 (0.85 - 2.73) | 0.157 | 1.86 (1.13 - 3.04) | 0.014 | 1.78 (1.12 - 2.82) | 0.014 |
| **Comorbidities^**^** |  |  |  |  |  |  |  |  |
| Asphyxia related disorders | - | - | 1 | - | 1 | - | 1 | - |
| Prematurity related disorders | 1 | - | - | - | 0.88 (0.67 - 1.15) | 0.338 | - | - |
| Infections related disorders | 1.21 (0.67 - 2.17) | 0.531 | 0.31 (0.22 - 0.45) | <0.001 | - | - | 0.306 (0.21 - 0.44) | <0.001 |
| Other disorders | 0.45 (0.25 - 0.83) | 0.010 | 0.73 (0.43 - 1.27) | 0.268 | 0.72 (0.42 - 1.25) | 0.242 | 0.75 (0.43 - 1.29) | 0.294 |
| **Apgar Score at 5 Minutes** |  |  |  |  |  |  |  |  |
| 10 (ref.) | 1 | - | 1 | - | 1 | - | 1 | - |
| 9 | 2.20 (0.48 - 9.93) | 0.307 | 1.84 (0.51 - 6.61) | 0.348 | 1.29 (0.34 - 4.84) | 0.710 | 2.15 (0.62 - 7.47) | 0.229 |
| 8 | 3.14 (0.69 - 14.33) | 0.139 | 1.93 (0.53 - 7.02) | 0.320 | 1.40 (0.37 - 5.27) | 0.615 | 2.58 (0.74 - 8.98) | 0.137 |
| 7 | 2.70 (0.59 - 12.35) | 0.199 | 1.96 (0.53 - 7.23) | 0.310 | 1.43 (0.38 - 5.36) | 0.592 | 2.47 (0.71 - 8.64) | 0.155 |
| 6 | 2.87 (0.62 - 13.31) | 0.178 | 3.73 (1.01 - 13.75) | 0.048 | 2.10 (0.56 - 7.90) | 0.270 | 3.87 (1.10 - 13.55) | 0.034 |
| 5 | 4.80 (1.04 - 22.22) | 0.045 | 3.94 (1.07 - 14.45) | 0.039 | 2.86 (0.76 - 10.66) | 0.119 | 4.86 (1.39 - 16.97) | 0.013 |
| 4 | 6.68 (1.39 - 32.18) | 0.018 | 3.49 (0.92 - 13.24) | 0.066 | 3.11 (0.81 - 11.95) | 0.099 | 5.38 (1.50 - 19.27) | 0.010 |
| ≤3 | 24.68 (5.46 - 111.63) | <0.001 | 13.81 (3.82 - 49.95) | <0.001 | 11.38 (3.08 - 42.05) | <0.001 | 20.10 (5.82 - 69.47) | <0.001 |
| **Level of Care** |  |  |  |  |  |  |  |  |
| Primary Level (ref.) | 1 | - | 1 | - | 1 | - | 1 | - |
| Secondary Level | 0.65 (0.20 - 2.14) | 0.480 | 0.68 (0.27 - 1.68) | 0.401 | 0.68 (0.30 - 1.51) | 0.338 | 0.68 (0.32 - 1.45) | 0.318 |
| Tertiary Level | 0.53 (0.16 - 1.74) | 0.296 | 0.41 (0.16 - 1.04) | 0.061 | 0.48 (0.21 - 1.09) | 0.078 | 0.46 (0.21 - 0.99) | 0.047 |
| * Comorbidities are classified according to the corresponding ICD-10 codes, as detailed in Table 1S.  Intergrowth Categories: Based on the Intergrowth-21st Project for assessing fetal and neonate growth. | | | | | | | | |
